# 2378. Use of Natural Language Processing to Extract Published Real World Data on a COVID Vaccine and Antiviral Treatment

**DOI:** 10.1093/ofid/ofad500.1999

**Published:** 2023-11-27

**Authors:** Anupama Raghuram, Anna Ssentongo, Lenon Mendes Pereira, Yuting Kuang, Jennifer Uyei, Peter Park

**Affiliations:** Pfizer, Louisville, Kentucky; IQVIA, Philadelphia, Pennsylvania; IQVIA, Philadelphia, Pennsylvania; IQVIA, Philadelphia, Pennsylvania; IQVIA, Philadelphia, Pennsylvania; Pfizer, Louisville, Kentucky

## Abstract

**Background:**

The COVID-19 global pandemic generated an exponential increase in scientific publications over the last 3 years. LitCovid includes ∼350,000 articles related to COVID-19, vaccine and antiviral medicine development, and real-world data (RWD). The need to identify and extract medical data from relevant articles presents an opportunity to use natural language processing (NLP) and machine learning to analyze large volumes of text related to COVID-19. We evaluated the use of NLP to rapidly identify and extract RWD from vaccine and antiviral effectiveness studies to summarize the data for healthcare professionals.

**Methods:**

We used a two-step approach comprised of (1) an automated NLP system with machine learning and rule-based methods to extract medical data from articles and (2) a manual evidence synthesis approach to confirm accuracy by expert review to create a RWD dataset for effectiveness related to BNT162b2 (Pfizer-BioNTech COVID-19 Vaccine) and nirmatrelvir/ritonavir (Paxlovid). NLP linguistic models for automatic data extraction were developed for topics related to vaccine effectiveness, boosters, fourth dose, variant-adapted vaccines, antiviral treatment effectiveness, and long COVID. RWD were captured into a novel data excel template.

**Results:**

NLP model training and development was conducted on CORD-19 Open Research Dataset, and NLP-based extraction has been applied on 100 studies. Elapsed NLP-based extraction time was compared to manual extraction by 6 scientific experts over a 10 day test, and speed gains of 2.5-3.0x were achieved. NLP-based and manual extraction of data from >130 publications was then used to present individual study results and summarize effectiveness results into two publicly available websites (www.vaccinemedicaldata.com; www.antiviralmedicaldata.com) with high data accuracy. NLP also allowed for automated labeling and output normalization of data into a scalable model using ontologies.

**Conclusion:**

NLP is an efficient technology for automated extraction of medical data from published literature for vaccine and antiviral effectiveness topics. Automated medical data mining using NLP may support end-to-end extraction of study results to quickly educate prescribers and improve vaccine and antiviral healthcare decision making.

**Disclosures:**

**Anupama Raghuram, MD**, Merck: Employee|Merck: Stocks/Bonds|Pfizer: Employee|Pfizer: Stocks/Bonds **Anna Ssentongo, PhD**, IQVIA: Employee **Lenon Mendes Pereira, PhD**, IQVIA: Employee **Yuting Kuang, PhD**, IQVIA: Employee **Jennifer Uyei, PhD, MPH**, IQVIA: Employee|IQVIA: Stocks/Bonds **Peter Park, PhD**, Pfizer: Employee|Pfizer: Stocks/Bonds

